# Cross-Omics Comparison of Stress Responses in Mesothelial Cells Exposed to Heat- versus Filter-Sterilized Peritoneal Dialysis Fluids

**DOI:** 10.1155/2015/628158

**Published:** 2015-10-01

**Authors:** Klaus Kratochwill, Thorsten O. Bender, Anton M. Lichtenauer, Rebecca Herzog, Silvia Tarantino, Katarzyna Bialas, Achim Jörres, Christoph Aufricht

**Affiliations:** ^1^Department of Pediatrics and Adolescent Medicine, Medical University of Vienna, 1090 Vienna, Austria; ^2^Zytoprotec GmbH, 1090 Vienna, Austria; ^3^Department of Nephrology and Medical Intensive Care, Campus Virchow-Klinikum, Charité Universitätsmedizin Berlin, 13353 Berlin, Germany

## Abstract

Recent research suggests that cytoprotective responses, such as expression of heat-shock proteins, might be inadequately induced in mesothelial cells by heat-sterilized peritoneal dialysis (PD) fluids. This study compares transcriptome data and multiple protein expression profiles for providing new insight into regulatory mechanisms. Two-dimensional difference gel electrophoresis (2D-DIGE) based proteomics and topic defined gene expression microarray-based transcriptomics techniques were used to evaluate stress responses in human omental peritoneal mesothelial cells in response to heat- or filter-sterilized PD fluids. Data from selected heat-shock proteins were validated by 2D western-blot analysis. Comparison of proteomics and transcriptomics data discriminated differentially regulated protein abundance into groups depending on correlating or noncorrelating transcripts. Inadequate abundance of several heat-shock proteins following exposure to heat-sterilized PD fluids is not reflected on the mRNA level indicating interference beyond transcriptional regulation. For the first time, this study describes evidence for posttranscriptional inadequacy of heat-shock protein expression by heat-sterilized PD fluids as a novel cytotoxic property. Cross-omics technologies introduce a novel way of understanding PDF bioincompatibility and searching for new interventions to reestablish adequate cytoprotective responses.

## 1. Introduction

Peritoneal dialysis (PD) is a cost effective and safe form of renal replacement therapy in end stage renal disease. However, PD-fluids (PDF) are bioincompatible solutions and may induce severe peritoneal damage, to a large part mediated by cytotoxic injury to the mesothelial cell layer, mostly due to low pH, lactate, high glucose, and its degradation products [1, 2].

In experimental PD we and others have shown that acute exposure to cytotoxic contents of PDF results in rapid induction of heat shock proteins (HSP) in mesothelial cells during the recovery phase, counteracting toxic injury [3–6]. HSP are the most prominent protein members of the cellular stress response and transient overexpression of these important molecules of the cellular repair machinery has been shown to mediate strong cytoprotective effects during experimental PD [5, 6].

Recently, we have described unexpectedly low HSP expression upon more extended exposure to diluted heat-sterilized PDF [7]. Albeit this setting still represents a highly artificial system, the exposure to diluted cytotoxic properties of PDF likely reflects intraperitoneal conditions during a PD dwell more closely than acute exposure to pure PDF [8–10]. Heat-sterilization and storage of glucose-based PDF result in formation of highly reactive glucose degradation products (GDPs) that are known to mediate their cytotoxicity via oxidative stress [11–13]. These findings suggest that exposure to PDF containing high levels of GDP may even dampen cellular stress responses, increasing the vulnerability of mesothelial cells against PDF cytotoxicity. Recent research suggests that oxidative stress might indeed suppress the cellular stress responses [14, 15].

In this study we have used two-dimensional difference gel electrophoresis (2D-DIGE) based proteomics and topic defined gene expression microarray-based transcriptomics techniques to evaluate mesothelial stress responses in response to exposure to heat- versus filter-sterilized PDF, thus comparing effects of GDPs on a global level. For the first time transcriptome data and multiple protein expression profiles were compared in experimental PD in order to provide new insight into regulatory mechanisms.

## 2. Materials and Methods

### 2.1. Materials

All chemicals, unless otherwise stated, were purchased from Sigma-Aldrich (St. Louis, MI, USA). All tissue culture plastics were Falcon (Becton Dickinson, San José, CA, USA). The PD solutions heat-sterilized PDF (H-PDF), containing GDPs, and filter-sterilized PDF (F-PDF), containing no GDPs, were prepared in the laboratory according to the following formulation: NaCl 5.786 g/L, CaCl_2_·2H_2_O 0.257 g/L, MgCl_2_·6H_2_O 0.102 g/L, sodium D/L-lactate 3.925 g/L, and anhydrous D-glucose 15.0 or 42.5 g/L, with a final composition in mmol/L: Na^+^ 132, Ca^2+^ 1.25, Mg^2+^ 0.25, Cl^−^ 95, lactate 40, and 3.86% glucose and a pH of 5.5. The solutions from the same stock were then sterilized either by heat (121°C, 0.2 MPa, 20 min) or by filtration through a 0.2-*μ*m pore size filter (Nalgene, Nalge Nunc International, Rochester, NY, USA).

### 2.2. Exposure of Human Peritoneal Mesothelial Cells to PD Solutions

Human peritoneal mesothelial cells (HPMC) were isolated from fully anonymized specimens of omentum obtained from three consenting nonuremic patients undergoing elective abdominal surgery. The study was accomplished in accordance with the institutional review board, consistent with the principles of the Declaration of Helsinki. Cells were isolated and characterized as previously described [16]. HPMC were propagated in M199 culture medium supplemented with 2 mM L-glutamine, 100 U/mL penicillin, 100 *μ*g/mL streptomycin, 0.4 *μ*g/mL hydrocortisone, and 10%_v/v_ fetal calf serum (FCS; Invitrogen, Carlsbad, CA, USA). All experiments were performed using cells from the second passage since later subcultures may contain an increasing number of senescent cells [16, 17]. HPMC were plated into multiwell clusters and grown until confluence. The standard medium containing 10% FCS was replaced by medium supplemented with 0.3% FCS for 48 hours prior to experiments to render the cells in a quiescent state.

*PDF Exposure*. In three independent experiments HPMC cultures obtained from the three above mentioned donors were exposed to a 1 : 1 mixture of regular culture medium containing 0.6% FCS (final concentration 0.3%) and the mentioned PD solution (either filter- or heat-sterilized) for 24 h. At the end of the exposure period the cells were harvested according to the procedure given below for 2D-DIGE and the supernatants were saved at −80°C until use in the viability assay (LDH release).

### 2.3. Protein Expression Profiling 2D-DIGE

#### 2.3.1. Protein Sample Preparation

The cells were lysed by incubation with 1 mL lysis buffer (30 mM Tris, pH 8.5, 7 M urea, 2 M thiourea, 4% 3-[(3-cholamidopropyl) dimethylammonio]-1-propanesulfonate (CHAPS), 1 mM EDTA, 1 tablet of Complete Protease Inhibitor (Roche, Basel; Switzerland) per 100 mL, and 10 *μ*L/mL of each of the phosphatase inhibitor cocktails 1 and 2 (Sigma-Aldrich)) per 3 × 10^7^ cells for 10 min at 25°C. The resulting lysates were centrifuged for 30 minutes (14,000 ×g, 4°C) and stored at −80°C until further processing. Total protein concentration was determined by the 2D-Quant Kit (GE Healthcare, Uppsala, Sweden) according to the manufacturer's manual.

#### 2.3.2. Cell Harvesting and Protein Labeling

An internal pooled standard (IPS) containing all sample pools was prepared and used in all gels. IPS therefore represents a mixture of all proteins expressed in any cell under all tested conditions and should thus contain every protein spot that can be detected. Aliquots of the samples (H-PDF, F-PDF) were each labeled with Cy5 as well as protein lysates from immortalized HPMC, which were used as reference material. Labeling of the IPS was performed with Cy3 dye using the DIGE minimal labeling kit (GE Healthcare) following the recommendations of the manufacturer with minor modifications. In brief, 40 *μ*g of total protein per sample was mixed with 200 pmol of the reconstituted Cy5 dye solution (400 *μ*M stock solution in anhydrous DMF) and per gel 40 *μ*g of total protein of the internal pooled standard (IPS) of all samples was mixed with 200 pmol of the reconstituted Cy3 CyDye solution. Labeling of the IPS was performed in one batch to achieve a uniform standard. The labeling reactions were incubated on ice in the dark for 30 min and then stopped with 1 *μ*L of 10 mM L-lysine solution. For every gel one Cy5 labeled sample and an aliquot of the Cy3 labeled IPS were mixed.

### 2.4. Isoelectric Focusing

The rehydration mix was brought to a final volume of 450 *μ*L with rehydration buffer consisting of 5 M urea, 0.5% CHAPS, 0.5% Pharmalyte, and 12 *μ*L/mL of DeStreak reagent (GE Healthcare). Each mixture was applied by rehydration loading to one IPG strip (ReadyStrip pH 3–10, nonlinear, 24 cm, Bio-Rad, Hercules, CA, USA) in the focusing tray of a Bio-Rad Protean IEF unit, sealed with silicone oil (Bio-Rad). The strips were rehydrated with the samples by “active rehydration” at 50 V and 20°C for 15 h and then focused for 3 h at 100 V, before the voltage was constantly increased to 8000 V within 17.5 hours, applying altogether 65 kVh with a maximum of 30 *μ*A per strip.

### 2.5. Vertical Electrophoresis

Gels for second-dimension vertical SDS-PAGE were cast using a Bio-Rad multicasting chamber, low-fluorescent glass plates, and 1 mm spacers (Bio-Rad). For a final concentration of the separation gels of 12%, 240 mL acrylamide stock solution (40%, T : C = 29 : 1, Bio-Rad) was mixed with 200 mL 1.5 M Tris-HCl pH 8.8, 40 mL glycerol, and 320 mL H_2_O_UHQ_. TEMED (80 *μ*L) and ammonium persulfate (1 mL, APS, 10% in H_2_O_UHQ_) were added after degassing of the mixture and right before filling of the casting chamber. The gels were left to polymerize overnight, overlaid with water-saturated n-butanol. Vertical second-dimension SDS-PAGE was carried out on a Bio-Rad Dodeca system with the current set to 60 mA for 100 Vh and then to 200 mA for 1200 Vh.

### 2.6. Fluorescence Image Acquisition and Data Analysis

DIGE labeled gels were scanned sandwiched between the low-fluorescent glass plates of the cassettes immediately after the run. Gel images were acquired using a Typhoon Trio laser scanner (GE Healthcare) using excitation and emission wavelengths recommended for the used dyes (Cy3: Ex 532 nm, Em 580 nm, and BP 30; Cy5: Ex 633 nm, Em 670 nm, and BP 30). The photomultiplier voltage was chosen so that the most abundant protein spots were close to saturation. Sensitivity level was set to “normal.”

Gel images were analyzed using the Delta2D 3.6 software (Decodon GmbH, Greifswald, Germany) using the algorithm designated for DIGE experiments. The images, containing the IPS, were aligned by pairwise warping and spot detection was carried out on a fused image of all gels (see Supplemental Figure  1 in Supplementary Material available online at http://dx.doi.org/10.1155/2015/628158). Protein identifications, accomplished in our laboratory [18], were processed with the aid of the ID mapping feature offered by the UniProt database (http://www.uniprot.org; [19]) to a short list of proteins overlapping with the genes investigated by RNA array used in this study (see Supplemental Table  1 for a summary of all used mass spectrometric identification data). The protein annotations of this short list were assigned to the respective spots on the 2D gels. Relative spot volumes normalized to the IPS of 28 unique proteins (see Table 1) were quantified among exposure of HPMC to H-PDF or F-PDF. Significance values were derived from group comparisons utilizing Student's* t*-test with the obtained *p* values given in Table 1. Details on individual proteins and corresponding spots are provided as bar graphs for each spot (Supplemental Figure  2) and spot album (Supplemental Figure  3).

### 2.7. RNA Expression Array Analysis

For analysis on the transcriptional level topic defined microarray experiments were employed. In brief, HPMC exposed to H-PDF or F-PDF were homogenized in 350 *μ*L RLT buffer (Qiagen, Hilden, Germany) and then extracted using the RNeasy Mini Kit (Qiagen) according to the manufacturer's protocol. Total RNA was checked for integrity with the Agilent Bioanalyzer 2100 (Agilent Technologies, Inc., Palo Alto, CA). 0.8 *μ*g of total RNA was then used for amplification and analysis with topic defined PIQOR Toxicology Human Microarray (Miltenyi Biotec, Bergisch Gladbach, Germany) containing 1264 human genes comprising the subject areas apoptosis, DNA damage and repair, inflammation, cell proliferation and response to oxidative stress, and xenobiotic metabolism. Each PIQOR microarray contains six housekeeping genes (ACTA2, CYPA, GAPDH, HPRT, TUBA, and TUBB) and six controls (herring sperm DNA, salt, and four artificial control RNAs) for the correct quantification of the differential expression patterns. Genes are spotted in quadruplicate. All steps between RNA isolation and data interpretation, including sample labelling, microarray hybridization, and scanning, were carried out by Miltenyi Biotec Microarray Services.

Data analysis included exclusion of low-quality spots, background subtraction to obtain the net signal intensity, data normalization, and calculation of the Cy5/Cy3 ratios. Additionally only spots that had at least in one channel a signal intensity that was 2-fold higher than the mean background were taken into account for the ratio calculation. Normalized mean Cy5/Cy3 ratios of the four replicates per gene and the respective coefficient of variation (% CV) were calculated. This CV refers to the average of the Cy5/Cy3 ratios for the gene replicates.

Finally, for the genes overlapping with identified proteins in the proteomics experiment mean ratios were calculated by averaging the values obtained from the three individual chips, one per biological experiment. The standard deviation (SD) and CV (Chip CV) are also given in the results (see Table 1).

### 2.8. Two-Dimensional Western Blotting (Adapted from [20])

For 2D western analysis, gels were prepared as described above. Proteins were electroblotted onto PVDF membranes (Millipore; Billerica, MA, USA) immediately after the run by semidry transfer using the Novablot unit of the MultiPhor II electrophoresis system and an according transfer buffer (200 mM glycine, 25 mM Tris base, 0.1% SDS, and 20% methanol). The membranes were washed in TBST buffer (150 mM NaCl, 0.05% Tween 20, and 10 mM Tris-HCl at pH 7.4) and stained using ruthenium II tris-bathophenanthroline disulfonate (RuBPS) following a fluorescent staining protocol, modified for staining membranes. In brief, RuBPS was prepared as published by Rabilloud et al. [21] and used as stock solution without further processing. The proteins were fixed by incubation for 15 min with fixing solution (10% acetic acid, 20% methanol). Membranes were washed 4 times for 5 min with H_2_O_UHQ_ and then incubated for 30 min with staining solution (10 *μ*L RuBPS stock solution made up to 1000 mL with H_2_O_UHQ_). After again washing 4 times for 5 min with H_2_O_UHQ_ the membranes were dried and scanned with the aid of the Typhoon Trio laser scanner mentioned above using excitation and emission wavelengths optimized for the used protocol (Ex 488 nm, Em 670 nm, and BP 30). The photomultiplier voltage was chosen so that the stained protein spots were clearly distinguishable from the background. Sensitivity level was set to “normal.” The obtained total protein pattern was used for later alignment to the specific immunodetected signals. The membranes were rehumidified with methanol and washed again in TBST buffer before proceeding to the blocking step. The membranes were blocked with 5% dry milk in TBST and then incubated with the primary murine antibody against Hsp72 (SPA-810, Stressgen/Assay Designs, Ann Arbor, MI, USA), Hsp27 (SPA-801, Stressgen/Assay Designs), or Hsp60 (SPA-806 Stressgen/Assay designs) dissolved in TBST containing 1% dry milk for 6 hours. After washing 3 times for 20 min in TBST and incubation with a secondary, peroxidase-coupled antibody (polyclonal rabbit anti-mouse Ig/HRP P0260, Dako Cytomation, Carpinteria, CA, USA) detection was accomplished by using enhanced chemiluminescence solution (Western Lightning Reagent, Perkin Elmer, Boston, MA, USA) and a ChemiDoc XRS chemiluminescence detection system (Bio-Rad).

## 3. Results

Exposure to H-PDF and F-PDF resulted in sublethal injury, evaluated by indiscernible cell density, assessed by light microscopy and cell counting, and comparable total protein concentrations of the cell lysates (H-PDF/F-PDF 76.6 ± 36.7% mean ± SD, *p* = 0.338). LDH release as a marker of loss of cellular membrane integrity was significantly higher following exposure to H-PDF than to F-PDF (H-PDF/F-PDF 887 ± 277% mean ± SD, *p* = 0.011).

For investigating potentially involved regulatory mechanisms both protein and RNA levels of HPMC undergoing treatment with H-PDF or F-PDF for 24 h were analyzed by 2D-DIGE and topic defined gene expression microarrays. In order to cover the largest possible number of transcripts and proteins we used all available mass spectrometric protein identifications in MC performed by our group until today [18, 22] and built a comprehensive 2D proteome map (see Supplemental Figure  1 and Supplemental Table  1). These identifications were screened for overlaps with the transcripts successfully quantified in the mRNA microarray. Of the 1264 genes contained on the microarray and the overall 185 protein identifications in mesothelial cells by mass spectrometry we could identify an overlap of 28 unique genes with the according proteins contained in 38 distinct spots (see Table 1, Supplemental Figure  2, and Supplemental Figure  3). The high reproducibility of the proteomics data obtained by 2D-DIGE was reflected by a low variability of the quantified spots (median CV was 9.7% for all protein spots contained in Table 1). The observation of more than one spot per protein is explained by the capability of this technique to detect individual isoforms or posttranslationally modified variants of the same protein. Exploration of the combined RNA and protein profiles allowed functional grouping of the analyzed candidates according to observed regulation patterns.

**Figure 1:**
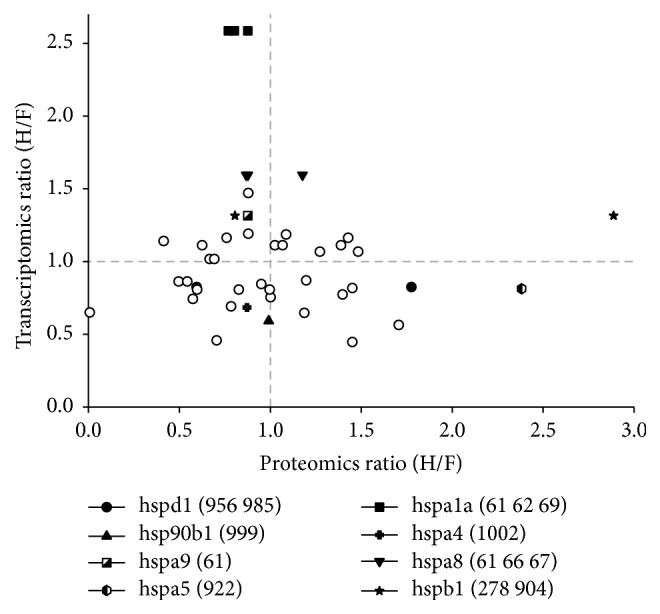
Cross-omics comparison of stress responses in mesothelial cells exposed to heat-versus filter-sterilized peritoneal dialysis fluid. HPMC cultures (*n* = 3) were continuously exposed to a 1 : 1 mix of heat- or filter-sterilized PDF (“H” or “F”) and cell culture medium for 24 hours. Data are expressed as ratio of the respective proteomics and transcriptomics results from heat- over those of filter-sterilized PDF exposed mesothelial cells (H/F). The comparison of proteomics and transcriptomics data allowed the discrimination of differentially regulated protein expression into groups depending on correlating or noncorrelating transcripts. The inadequate expression of several HSP (full symbols) on the protein level is not reflected on the transcriptional level indicating potential interference of GDPs in translational activity and regulation.

When grouping these protein spots according to their expression on the protein and RNA level by calculation of a H-PDF/F-PDF ratio, four discrete groups could be built (see Figure 1). Thirteen spots had a protein ratio below 1.0, meaning less protein abundance when exposed to H-PDF, whereas their RNA ratio was above 1.0. Seven of these spots (54%) contained HSP (protein symbols: HSPA9, HSPA1A, HSPA8, HSPA1A, HSPA8, HSPA1A, G6PD, GSTP1, CCT2, TXNRD1, G6PD, HSPB1, and NME1). Six protein spots showed a protein ratio above 1.0 with simultaneously elevated RNA expression (>1.0). Two of these spots contained HSP (HSPA8, HSPB1, GSTP1, GSR, PDIA3, and PDIA3). However, other protein isoforms of these two HSP (HSPA8, HSPB1) were also contained in the previously mentioned group. Eight protein spots showed higher abundance on the protein level although their RNA expression ratio was below 1.0. Two of these spots contained HSP (CCT5, PSMB2, HSPA5, P4HB, PDIA6, COPS4, HSPD1, and RPSA). Eleven protein spots had a H-PDF/F-PDF ratio of less than 1.0 with concomitantly downregulated RNA expression. Three of these spots contained HSP (CCT7, PSMA2, FKBP1A, PPIA, PCNA, HADHA, PPIA, PSMD4, HSPD1, HSP90B1, and HSPA4). In addition to the graphical presentation in Figure 1, numerical values of RNA expression ratios as well as spot abundance data under the experimental conditions of extended heat- versus filter-sterilized PDF treatment are given in Table 1 together with their statistical parameters and *p* values indicating significant changes. Additional bioinformatics analysis of the transcriptomics data only found enrichment of biological processes attributable to immune response, angiogenesis, injury/repair mechanism, and apoptosis (see Supplemental Table  2).

**Figure 2:**
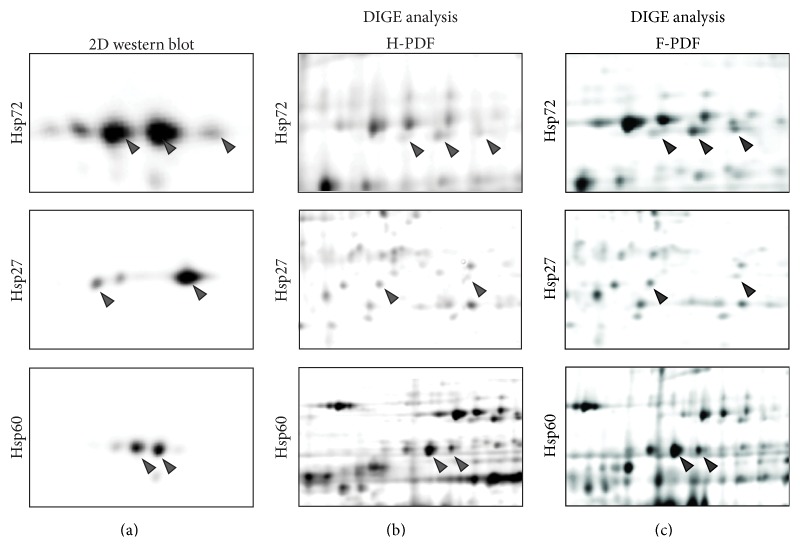
Two-dimensional western analysis of the chaperones Hsp70, Hsp27, and Hsp60. (a) shows the result of the immunoblots with positive signals for the respective specific antibodies given in each line and the MS identified protein spots indicated by grey arrow heads. For immunodetection of all relevant spots, pooled samples were used in order to represent all relevant isoforms and modifications. (b, c) show the identical regions in the DIGE gels, where the middle panel shows the protein separation of total cell extracts from mesothelial cells following exposure to H-PDF, and (c) shows the protein separation of total cell extracts from mesothelial cells following exposure to F-PDF. Again the MS identified protein spots are indicated by grey arrow heads.

In Figure 2, results of 2D western blotting are shown for the protein spots of prototypical members of the major HSP families, that is, Hsp72 (HSPA1A), Hsp27 (HSPB1), and Hsp60 (HSPD1), demonstrating that the mass spectrometric data on protein identities might not cover all isoforms that are recognized by specific antibodies. Whereas the antibody-based detection might yield unspecific signals, the spot identities that were confirmed by both western blotting and mass spectrometric data represent very robust information.

## 4. Discussion

Comparing the effects of two PDF that only differ in their modality of sterilization, either by heat or by filtration, can be regarded as surrogate method to evaluate the effects of GDPs formed through heat-sterilization [23]. Specific effects of heat-sterilized PDF on the cellular stress response might thus be largely attributed to these toxic compounds. GDPs are known to mediate their cytotoxicity via oxidative stress, and recent research suggests that oxidative stress might dampen the cellular stress responses [14, 15].

The advent of omics technologies, such as gel- or mass spectrometry-based proteomics of the protein level and microarray techniques on the transcriptional level, allowed unbiased global analyses, searching for yet unknown differentially regulated proteins or transcripts under given experimental conditions.

Whereas the use of a single analytical level allows candidate search and functional interpretation based on common features, for example, characterized in gene ontology databases, the cross-omics approach allows generating hypotheses by comparing gene expression profiling to (functional active) protein abundances. In this study we used a topic defined mRNA microarray for toxicology relevant genes, provided by Miltenyi as the PIQOR platform. In contrast to quantification of mRNA levels by rt-PCR, which relies on the consistency of single housekeeping genes, the array technique allows more elaborate approaches of normalization.

Together with our proteomics platform based on 2D-DIGE we compared effects of heat- versus filter-sterilized PDF in mesothelial cells in the extended exposure model, for proteins identified by mass spectrometry in earlier* in vitro* PD studies.

The high relevance of the chosen candidate proteins and transcripts becomes evident by the considerable overlap of MS identified proteins and genes represented on the PIQOR array in experimental PD. We could therefore use this set, mainly consisting of chaperones and stress-relevant proteins involved in detoxification and protein homeostasis, to search for global systematic effects of heat- versus filter-sterilized PDF on the cellular stress response. In recent work, we have shown at the protein level that expression levels of such a stress proteome can be related to mesothelial cell susceptibility to PDF induced injury [24]. This stress proteome was found to be downregulated following exposure to heat-sterilized PDF and could be restored by addition of cytoprotective additives, such as alanyl-glutamine dipeptide [24]. The cross-omics approach used in the current study gives additional information beyond the observed inadequate induction of the heat shock response at the protein level [7, 24].

In our dataset a rather large group of candidates showed a protein abundance ratio below 1.0 (downregulated by H-PDF compared to F-PDF) and at the same time an mRNA expression ratio above 1.0 (upregulated by H-PDF compared to F-PDF), when the two fluid types were compared. This phenomenon is of growing interest in the omics-field, as the observed perturbations might allow deducing regulatory mechanisms [25]. Future studies are needed to investigate whether the reverse relationship of protein and mRNA indicates a higher degree of protein turnover (i.e., shortened half-life of the protein by increased degradation) or translational inhibition or posttranslational modifications [26, 27]. This group contains many HSP but the effect only reached the level of significance for the proteins Hsp72 (HSPA1A) and glucose-6-phosphate dehydrogenase (G6PD), which might be due to inherent limited power of these hypotheses-generating omics studies. HSP are molecular chaperones and known to incorporate transport and folding of other proteins by binding to hydrophobic normal hidden domains of immature or denatured proteins. Thereby, these proteins have been shown to promote cytoskeletal repair and preserve the mesothelial monolayer [5, 6]. Whereas G6PD was already found upregulated in the acute exposure model in an earlier study [28] this key-enzyme of the pentose phosphate pathway, which is substantial for the production of NADPH and the cell's resistance to oxidative stress [29, 30], is significantly downregulated by heat-sterilized PDF compared to filter-sterilized PDF after extended exposure. The initial observation that the cells are not able to adequately respond to stress mediated by toxic factors of PDF, such as glucose and GDPs, is further reflected by the fact that all candidates in the dataset belonging to the glutathione system (GSTP1, TXNRD1, and GSR), which is dedicated to detoxification and neutralization of reactive oxygen species, are found to be upregulated on the transcriptional level. However, none of these candidates show significant upregulation of protein abundance. The deleterious effect of the cooccurrence of hyperglycemia, toxic aldehydes, such as GDPs, and a lack of reductive power (e.g., by NADPH and glutathione) has been demonstrated extensively in the diabetes model and beyond [31–33].

Two further subgroups of our dataset were characterized by either simultaneously increased or decreased abundance of both protein and mRNA. Concurrent regulation of protein and mRNA likely indicates undisturbed translation of transcriptional regulation of gene expression into proteins.

One clearly upregulated player identified in our study is PDIA3, a protein disulfide-isomerase predominantly found in the endoplasmic reticulum lumen, which is involved in the unfolded protein response [34]. Interestingly, knockdown of PDIA3, also known as endoplasmic reticulum resident protein 57 or 58 kDa glucose-regulated protein, protected against tunicamycin-induced apoptosis, with associated induction of the 78 kDa glucose-regulated protein (GRP-78, BiP, and HSPA5) [35], which was identified as downregulated on the transcriptional level but upregulated on the protein level in our study. GRP-78 is an ER stress protein (glucose-regulated protein) and, as suggested by its name, expected to be upregulated by the physicochemical properties of PDF.

Another protein found with significantly higher abundance, when H-PDF was compared to F-PDF, was Hsp27 (HSPB1) which is well known to be involved in the stress response to PDF [3, 4]. Hsp27 is a highly abundant effector of the heat shock response directly interacting with the actin cytoskeleton and thereby protecting mesothelial cell integrity by increased abundance [36]. However, it is well known that Hsp27 is extensively modified by phosphorylation leading to reciprocal abundance changes of multiple spots on 2D gels [36, 37]. Indeed we also identified Hsp27 in a spot with lowered abundance, as can be better made evident by direct comparison between 2D proteomic gels and 2D western analysis. As the phosphorylation status is functionally highly relevant for its chaperoning effect, Hsp27 is a particularly informative candidate to demonstrate the role of posttranslational modification on the level of functionally active protein isoforms versus total protein abundance and gene expression.

One clearly downregulated player, on the protein level as well as on the transcriptional level, identified in our study is PPIA which is a peptidyl-prolyl cis-trans-isomerase and is thereby active in protein folding [38]. PPIA is also a member of the immunophilin family (cyclophilin A) [39] and the receptor for the immunosuppressive drugs tacrolimus and cyclosporine [40, 41]. Other examples for proteins with significantly downregulated transcripts were the important ER chaperone Hsp60 (HSPD1), which was as Hsp27 also found in a significantly upregulated spot, and the proliferating cell nuclear antigen (PCNA), which is also known as cyclin, reflecting an increase in MC proliferation as previously described in the* in vivo* setting [42].

A small group of candidates showed a protein abundance ratio above 1.0 (upregulated by H-PDF compared to F-PDF) and at the same time an mRNA expression ratio below 1.0 (downregulated by H-PDF compared to F-PDF), when the two fluid types were compared. In this case the increased levels of protein expression with concomitantly depressed transcription of the same genes allow generating interesting hypotheses, such as prolonged half-life of the proteins by decreased degradation, possibly mediated by lack of protein degrading mechanisms such as the ubiquitin-proteasome pathway [26].

Interestingly all identified members associated with the ubiquitin-proteasome pathway (PSMB2, COPS4, PSMA2, and PSMD4) showed downregulated levels of mRNA and one of them was also significantly lower abundant on the protein level. This effect is concordant with the literature, where hyperglycemia and methylglyoxal led to an impaired ubiquitin-proteasome pathway in bovine and murine endothelial cells [32]. Indeed methylglyoxal is one of the GDPs detected in considerable concentrations in conventional PDF [43]. In this study nonuremic patients were used to obtain primary mesothelial cells from omentum. It has been demonstrated before that the uremic milieu per se can change the behavior of the mesothelial cells. Thus, future studies are needed to investigate whether specific GDPs in PDF might impair or deplete this essential part of the cellular stress response and what the role of the uremic milieu with even more toxic small molecules might be [44, 45].

Our data show that under stressful conditions the correlation between mRNA and protein cannot be regarded as linear for a wide range of tightly involved players of the stress response. The transcription of mRNA is the initial level of gene regulation, where transcription factors lead to situation-dependent usage of genetic sequence. However a plethora of intermitting mechanisms, such as RNA interference, regulatory proteins, or translational efficiency, can promote or hinder cellular protein production. Finally the amounts of functional proteins are influenced by protein folding, posttranslational modification, and turnover [26, 27]. Albeit the observed changes on the protein level are quite low, they are in a reasonable biological range, given that the proteins that can be detected by 2D gel electrophoresis represent the most abundant portion of soluble proteins. These findings highlight the limitations of gene expression profiling concerning the prediction of abundance of functionally active proteins and/or their isoforms. Future studies need to carefully assess these regulatory mechanisms to monitor the abundance of effector proteins that ultimately reflect biological reality.

Bioinformatics analysis yields similar information on activated stress responses using data derived from this transcriptomics dataset as we have previously reported in a proteomics approach [22]. Direct comparison between these “omics” technologies at the level of individual gene products, however, as we performed in our cross-omics approach, might allow additional interesting insights into specific pathogenic processes caused by PDF exposure. Moreover, the results of the current study underline that information of potential diagnostic (such as biomarker candidates) and/or therapeutic (such as novel drug targets) implications derived from proteomics and/or transcriptomics findings cannot be utilized interchangeably but rather request specific separate analysis and interpretation.

Taken together, the comparison of proteomics and transcriptomics data allowed the discrimination of differentially regulated protein expression according to correlating or noncorrelating transcripts. The results of this study are particularly interesting in terms of limitations of gene expression profiling with regard to prediction of abundance of functionally active proteins, indicating the need for future studies to investigate potential interference in translational activity and regulation.

## Supplementary Material

The supplementary material contains details of the 2D-DIGE expression profiling and bioinformatic analysis. Supplemental Figure 1 contains an identification map of protein spots used for the cross-omics comparison as well as the individual full-scale images of the sample channel. Supplemental Figure 2 provides details on abundance of investigated proteins, such as bar-charts of the mean spot abundance in each group. Supplemental Figure 3 provides details on investigated protein spots. Supplemental Table 1 contains details on protein identifications in spots used for cross-omics comparison. Supplemental Table 2 provides additional information on the analysis of the topic-defined microarray.

## Figures and Tables

**Table 1:** Proteins and transcripts analyzed by 2D-DIGE and mRNA expression microarrays.

Gene name	Gene ID	Protein name	Transcriptomics data					Proteomics data						
			RefSeq	Heat/filter				SwissProt entry name	Heat		Filter		Heat/filter	
				Mean ratio^(1)^	SD^(2)^	CV^(3)^	Chip CV^(4)^		Mean spot volume^(5)^	SD^(6)^	Mean spot volume^(5)^	SD^(6)^	Ratio^(7)^	*p* value^(8)^
*Increased protein abundance (proteomics ratio > 1)*														

Increased gene expression (transcriptomics ratio > 1)														
HSPA8	3312	Heat shock cognate 71 kDa protein	NM_006597 NM_153201	1.60	0.02	3%	4%	HSP7C_HUMAN	132.893	9.736	112.899	11.838	1.177	0.203
HSPB1	3315	Heat shock protein beta-1^*∗*^	NM_001540	1.32	0.13	17%	4%	HSPB1_HUMAN	207.830	35.162	71.975	17.335	2.888	0.061
GSTP1	2950	Glutathione S-transferase P	NM_000852	1.16	0.04	5%	4%	GSTP1_HUMAN	88.029	66.625	61.654	10.343	1.428	0.562
GSR	2936	Glutathione reductase, mitochondrial	NM_000637	1.19	0.07	9%	0%	GSHR_HUMAN	140.743	12.299	129.512	4.931	1.087	0.438
PDIA3	2923	Protein disulfide-isomerase A3^*∗∗*^	NM_005313	1.07	0.14	14%	14%	PDIA3_HUMAN	185.997	3.950	125.444	20.051	1.483	0.031
PDIA3	2923	Protein disulfide-isomerase A3^*∗*^	NM_005313	1.07	0.14	14%	14%	PDIA3_HUMAN	147.417	4.727	115.852	16.524	1.272	0.094

Decreased gene expression (transcriptomics ratio < 1)														
CCT5	22948	T-complex protein 1 subunit epsilon^*∗∗*^	NM_012073	0.82	0.09	7%	9%	TCPE_HUMAN	177.988	10.033	122.697	8.415	1.451	0.019
PSMB2	5690	Proteasome subunit beta type-2	NM_002794	0.87	0.09	7%	18%	PSB2_HUMAN	100.167	7.492	83.670	13.559	1.197	0.161
HSPA5	3309	78 kDa glucose-regulated protein^*∗∗∗*^	NM_005347	0.81	0.14	11%	4%	GRP78_HUMAN	254.121	5.404	106.676	15.450	2.382	0.001
P4HB	5034	Protein disulfide-isomerase^*∗∗*^	NM_000918	0.56	0.35	20%	7%	PDIA1_HUMAN	192.710	6.746	112.938	26.334	1.706	0.025
PDIA6	10130	Protein disulfide-isomerase A6^*∗*^	NM_005742	0.65	0.04	2%	4%	PDIA6_HUMAN	125.940	2.434	106.148	11.478	1.186	0.090
COPS4	51138	COP9 signalosome complex subunit 4	NM_016129	0.77	0.11	8%	7%	CSN4_HUMAN	151.484	17.903	108.440	17.368	1.397	0.139
HSPD1	3329	60 kDa heat shock protein, mitochondrial^*∗∗∗*^	NM_002156 NM_199440	0.82	0.06	5%	3%	CH60_HUMAN	275.662	1.224	155.233	8.962	1.776	0.001
RPSA	3921	40S ribosomal protein SA	NM_002295	0.75	0.24	18%	0.05	RSSA_HUMAN	111.019	23.693	110.845	9.474	1.002	0.993

*Decreased protein abundance (proteomics ratio < 1)*														

Increased gene expression (transcriptomics ratio > 1)														
HSPA9	3313	Stress-70 protein, mitochondrial	NM_004134	1.32	0.07	9%	4%	GRP75_HUMAN	108.049	9.256	123.253	5.843	0.877	0.156
HSPA1A	3303	*Heat shock 70 kDa protein 1*	NM_005345 NM_005346	2.59	0.07	17%	6%	HSP71_HUMAN	108.049	9.256	123.253	5.843	0.877	0.156
HSPA8	3312	Heat shock cognate 71 kDa protein	NM_006597 NM_153201	1.60	0.02	3%	4%	HSP7C_HUMAN	108.049	9.256	123.253	5.843	0.877	0.156
HSPA1A	3303	Heat shock 70 kDa protein 1^*∗*^	NM_005345 NM_005346	2.59	0.07	17%	6%	HSP71_HUMAN	104.254	14.587	130.041	4.812	0.802	0.091
HSPA8	3312	Heat shock cognate 71 kDa protein	NM_006597 NM_153201	1.60	0.02	3%	4%	HSP7C_HUMAN	106.853	10.219	123.120	6.412	0.868	0.163
HSPA1A	3303	Heat shock 70 kDa protein 1^*∗∗*^	NM_005345 NM_005346	2.59	0.07	17%	6%	HSP71_HUMAN	87.591	12.214	113.858	5.948	0.769	0.043
G6PD	2539	Glucose-6-phosphate 1-dehydrogenase^*∗∗*^	NM_000402	1.02	0.08	8%	6%	G6PD_HUMAN	80.836	16.641	121.330	10.229	0.666	0.035
GSTP1	2950	Glutathione S-transferase P	NM_000852	1.16	0.04	5%	4%	GSTP1_HUMAN	65.268	7.174	85.964	20.885	0.759	0.190
CCT2	10576	T-complex protein 1 subunit beta	NM_006431	1.19	0.03	3%	2%	TCPB_HUMAN	104.714	6.378	119.187	12.088	0.879	0.267
TXNRD1	7296	Thioredoxin reductase 1, cytoplasmic	NM_001093771 NM_003330 NM_182729 NM_182742 NM_182743	1.47	0.12	17%	6%	TRXR1_HUMAN	104.714	6.378	119.187	12.088	0.879	0.267
G6PD	2539	Glucose-6-phosphate 1-dehydrogenase^*∗∗∗*^	NM_000402	1.02	0.08	8%	6%	G6PD_HUMAN	85.848	8.356	124.140	5.506	0.692	0.005
HSPB1	3315	Heat shock protein beta-1	NM_001540	1.32	0.13	17%	4%	HSPB1_HUMAN	69.930	23.921	86.813	8.060	0.806	0.258
NME1	4830	Nucleoside diphosphate kinase A	NM_000269 NM_198175	1.14	0.08	9%	8%	NDKA_HUMAN	59.789	20.657	144.627	66.411	0.413	0.283

Decreased gene expression (transcriptomics ratio < 1)														
CCT7	10574	T-complex protein 1 subunit eta	NM_006429	0.81	0.04	3%	2%	TCPH_HUMAN	95.381	7.459	95.731	10.280	0.996	0.969
PSMA2	5683	Proteasome subunit alpha type-2	NM_002787	0.69	0.15	10%	4%	PSA2_HUMAN	116.477	11.780	148.569	14.466	0.784	0.150
FKBP1A	2280	Peptidyl-prolyl cis-trans-isomerase FKBP1A	NM_054014	0.65	0.26	17%	15%	FKB1A_HUMAN	473.565	141.421	65245.936	99.946	0.007	0.275
PPIA	5478	Peptidyl-prolyl cis-trans-isomerase A^*∗∗∗*^	NM_021130	0.86	0.04	4%	6%	PPIA_HUMAN	60.340	16.561	121.603	9.785	0.496	0.005
PCNA	5111	Proliferating cell nuclear antigen^*∗∗∗*^	NM_002592 NM_182649	0.46	0.26	12%	7%	PCNA_HUMAN	87.296	8.040	123.909	6.426	0.705	0.008
HADHA	3030	Trifunctional enzyme subunit alpha, mitochondrial	NM_000182	0.85	0.05	4%	5%	ECHA_HUMAN	85.564	23.723	90.073	12.598	0.950	0.798
PPIA	5478	Peptidyl-prolyl cis-trans-isomerase A^*∗∗∗*^	NM_021130	0.86	0.04	4%	6%	PPIA_HUMAN	62.919	11.686	115.738	10.498	0.544	0.006
PSMD4	5710	26S proteasome non-ATPase regulatory subunit 4^*∗∗∗*^	NM_002810 NR_002319	0.74	0.09	7%	8%	PSMD4_HUMAN	85.455	14.214	148.812	10.063	0.574	0.010
HSPD1	3329	60 kDa heat shock protein, mitochondrial^*∗∗∗*^	NM_002156 NM_199440	0.82	0.06	5%	3%	CH60_HUMAN	101.423	11.218	170.552	7.472	0.595	0.005
HSP90B1	7184	Endoplasmin	NM_003299	0.59	0.19	11%	5%	ENPL_HUMAN	92.818	33.376	93.651	22.914	0.991	0.977
HSPA4	3308	Heat shock 70 kDa protein 4	NM_002154	0.68			4%	HSP74_HUMAN	123.829	30.682	141.986	16.324	0.872	0.595

Proteins and transcripts, which could be mapped and analyzed by both proteomics by 2D-DIGE combined with MS protein identification and topic defined mRNA expression microarray analysis (Miltenyi PIQOR toxicology array). ^(1)^Ratio: fold-change of mRNA expression of cells treated with H-PDF versus cells treated with F-PDF as obtained from the PIQOR microarray service. ^(2)^Standard deviation of mRNA expression ratios of cells treated with H-PDF versus cells treated with F-PDF. ^(3)^Coefficient variation: relative standard deviation in percent of mRNA expression ratios of cells treated with H-PDF versus cells treated with F-PDF. ^(4)^Chip CV: the column contains the relative standard deviation in percent for the respective rRNA by the number of multiple features on the microarray (*n* = 4) as obtained from the PIQOR microarray service. ^(5)^Mean spot volume: mean of the spot quantification data over the biological replicates within the given groups.   ^(6)^Standard deviation of the spot quantification data within the given groups. ^(7)^Ratio: fold-change of protein spot abundance in cells treated with H-PDF versus cells treated with F-PDF as obtained from 2D-DIGE analysis. ^(8)^The column contains the *p* value of the *t*-test comparing relative spot abundance of cells treated with H-PDF versus cells treated with F-PDF. Protein quantifications, reaching the according levels of statistical significance, are marked with asterisks (^*∗*^ *p* < 0.1, ^*∗∗*^ *p* < 0.05, and ^*∗∗∗*^ *p* < 0.01) after the protein name.
