# Supplementary material for: Cross-Omics Comparison of Stress Responses in Mesothelial Cells Exposed to Heat- versus Filter-Sterilized Peritoneal Dialysis Fluids
Source: Biomed Res Int. 2015 Oct 1;2015:628158. doi: 10.1155/2015/628158 (PMC4606138; doi:10.1155/2015/628158)
Supplement: Supplementary file 1 — The supplementary material contains details of the 2D-DIGE expression profiling and bioinformatic analysis. Supplemental Figure 1 contains an identification map of protein spots used for the cross-omics comparison as well as the individual full-scale images of the sample channel. Supplemental Figure 2 provides details on abundance of investigated proteins, such as bar-charts of the mean spot abundance in each group. Supplemental Figure 3 provides details on investigated protein spots. Supplemental Table 1 contains details on protein identifications in spots used for cross-omics comparison. Supplemental Table 2 provides additional information on the analysis of the topic-defined microarray. [file 628158.f1.pdf]

Supplemental Figure 1

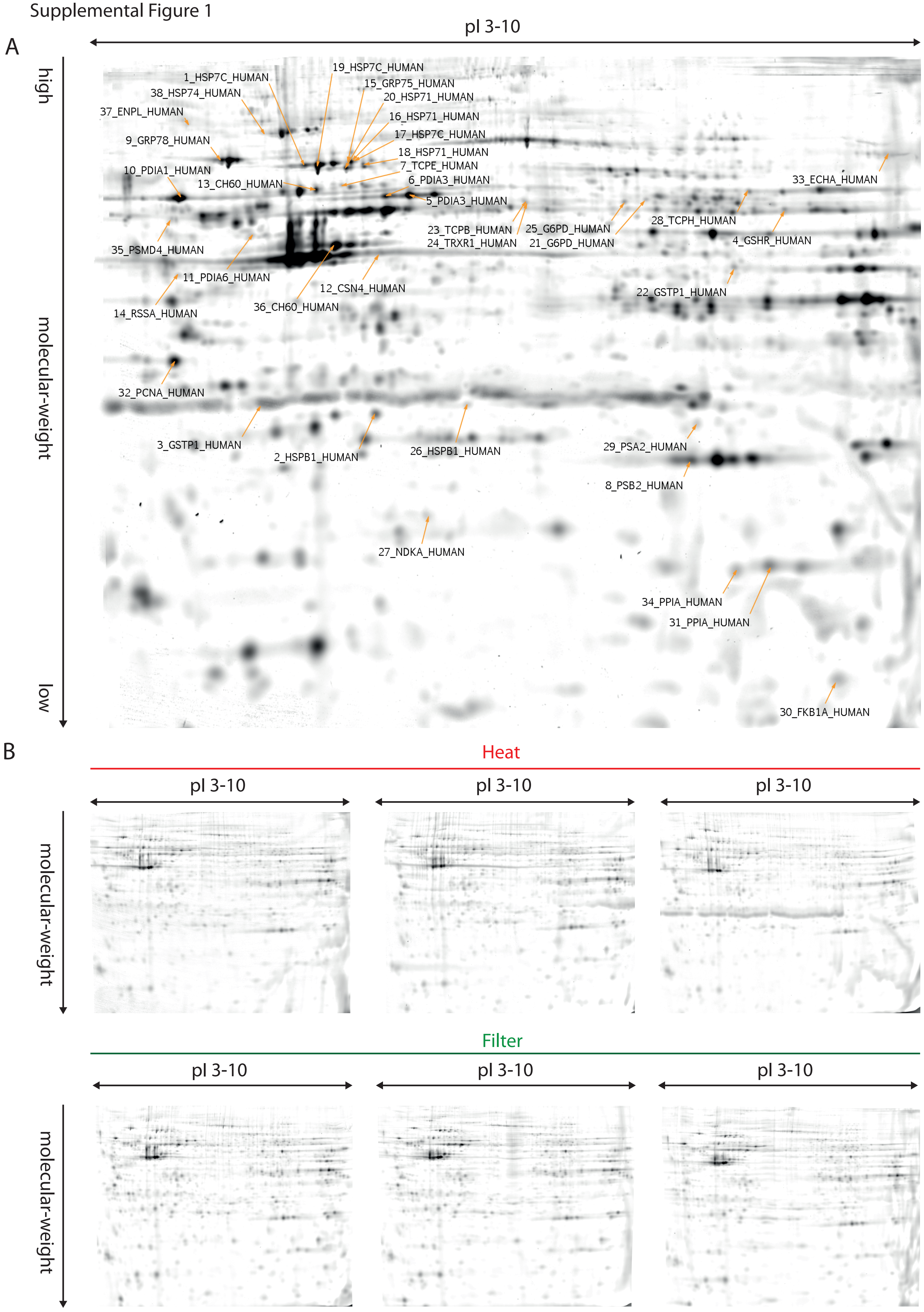

Legend to Supplemental Figure 1: Protein identification map an full-scale 2D gel images.

Panel A contains an identification map of protein spots used for the cross-omics comparison based on a fusion of the sample images, generated by the 2D gel analysis software (Delta 2D 3.4, Decodon, Greifswald, Germany). Panel B gives the individual full-scale images of the Cy3 sample channel.

Supplemental Figure 2

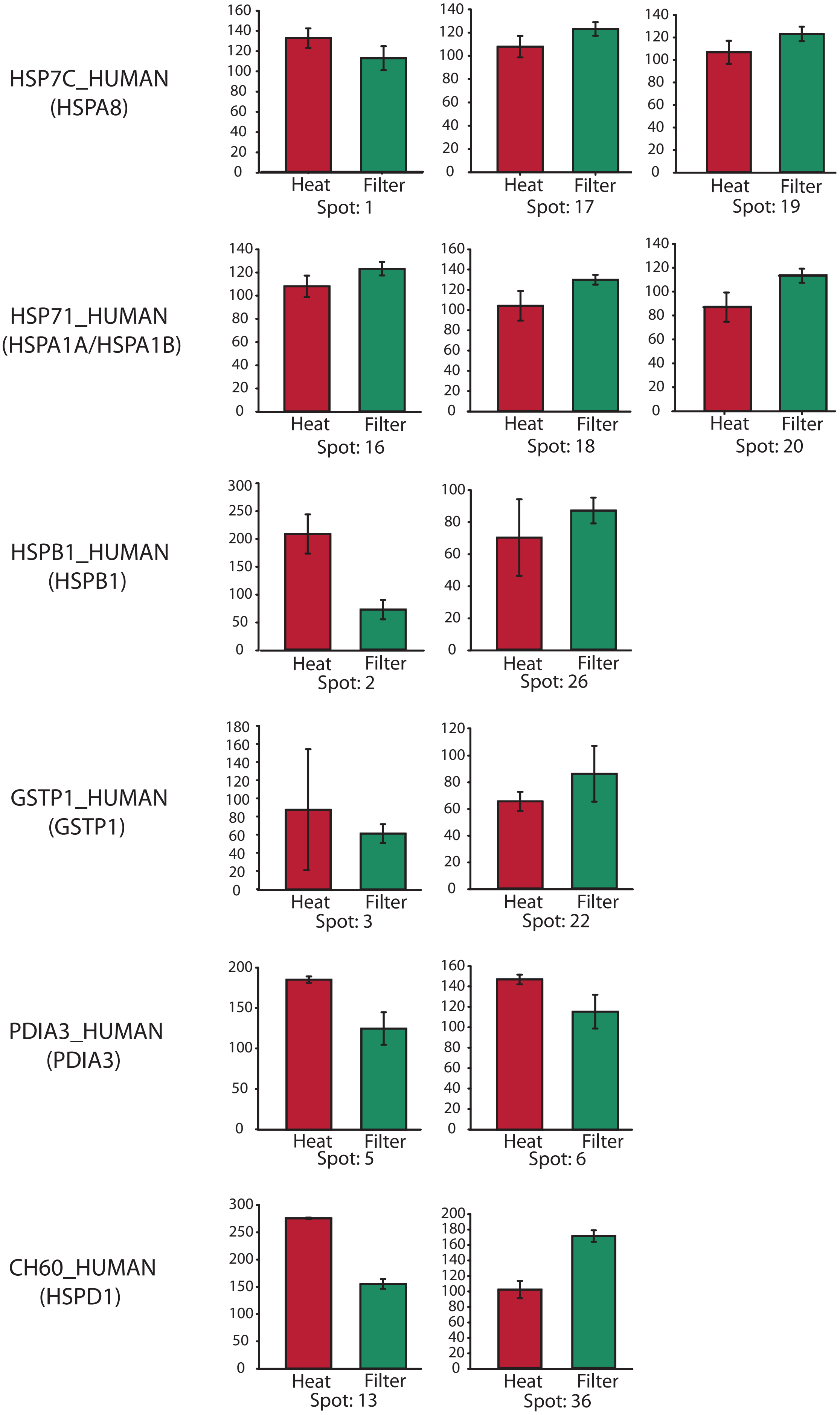

figure continued on next page

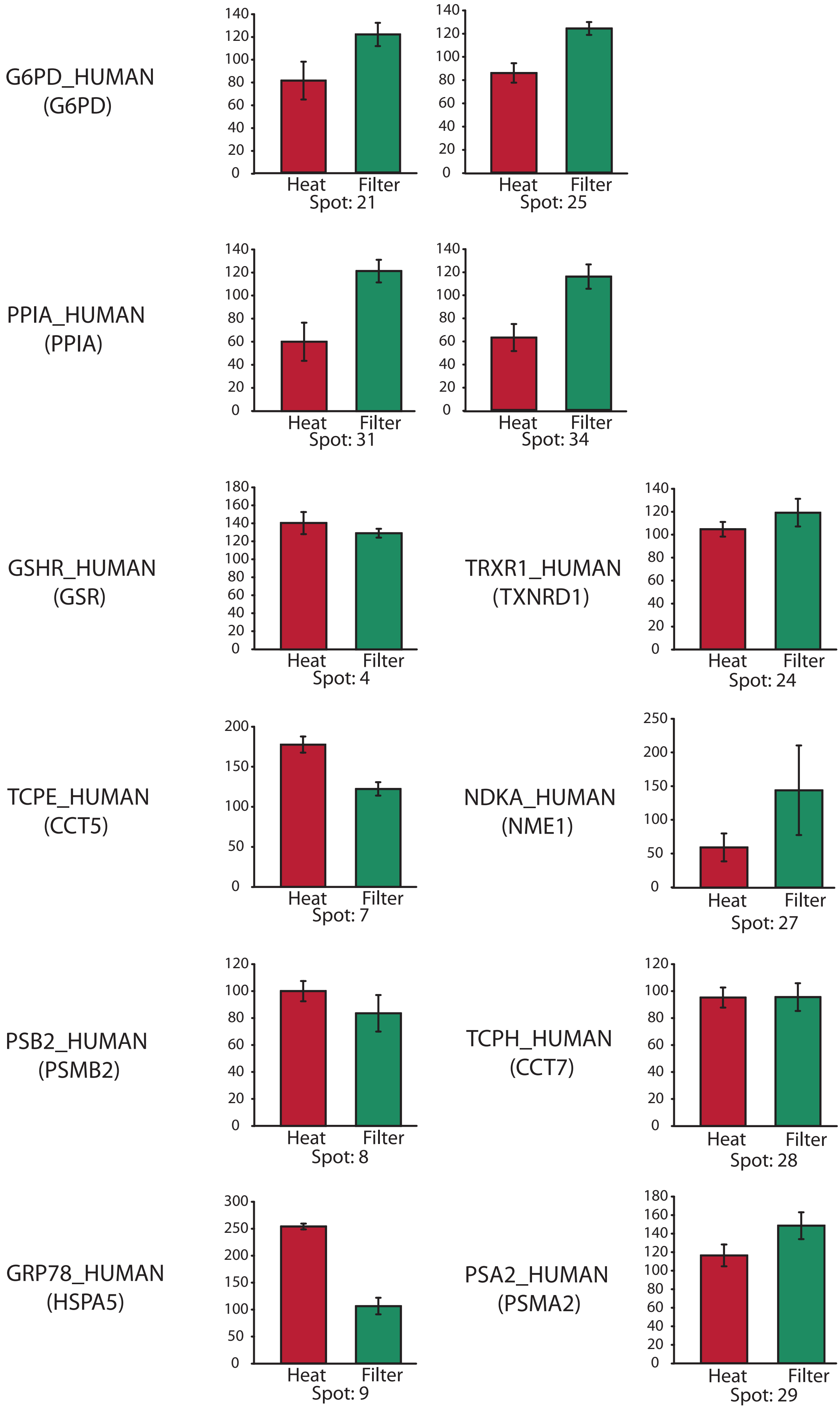

figure continued on next page

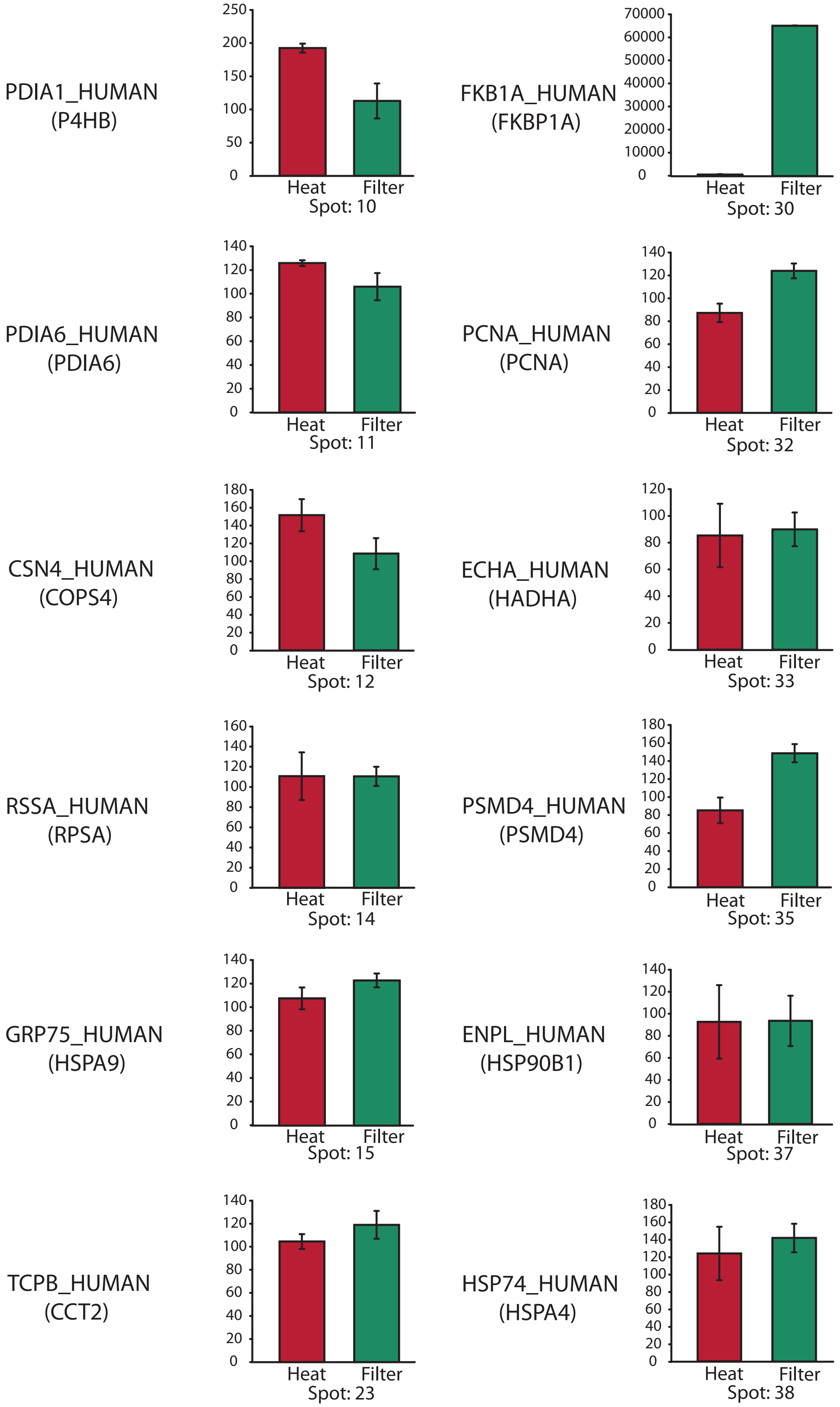

PDIA1_HUMAN
(P4HB)
250
200
150
100
50
0
Heat
Filter
Spot: 10
FKB1A_HUMAN
(FKBP1A)
70000
60000
50000
40000
30000
20000
10000
0
Heat
Filter
Spot: 30
PDIA6_HUMAN
(PDIA6)
140
120
100
80
60
40
20
0
Heat
Filter
Spot: 11
PCNA_HUMAN
(PCNA)
140
120
100
80
60
40
20
0
Heat
Filter
Spot: 32
CSN4_HUMAN
(COPS4)
180
160
140
120
100
80
60
40
20
0
Heat
Filter
Spot: 12
ECHA_HUMAN
(HADHA)
120
100
80
60
40
20
0
Heat
Filter
Spot: 33
RSSA_HUMAN
(RPSA)
160
140
120
100
80
60
40
20
0
Heat
Filter
Spot: 14
PSMD4_HUMAN
(PSMD4)
180
160
140
120
100
80
60
40
20
0
Heat
Filter
Spot: 35
GRP75_HUMAN
(HSPA9)
140
120
100
80
60
40
20
0
Heat
Filter
Spot: 15
ENPL_HUMAN
(HSP90B1)
140
120
100
80
60
40
20
0
Heat
Filter
Spot: 37
TCPB_HUMAN
(CCT2)
140
120
100
80
60
40
20
0
Heat
Filter
Spot: 23
HSP74_HUMAN
(HSPA4)
180
160
140
120
100
80
60
40
20
0
Heat
Filter
Spot: 38

Legend to Supplemental Figure 2: Details on abundance of investigated proteins.

The column giving the SwissProt protein ID is followed by a bar-chart representing on the y-axis the mean spot abundance in each group as normalized percent volume relative to the internal pooled standard (IPS). The protein symbol is given in parentheses underneath the SwissProt protein ID (*e.g.* the symbol for the gene HSPA1A / HSPA1B (SwissProt ID HSP71_HUMAN) represents what is commonly known as Hsp72). The actual abundance of each DIGE replicate results from the relative spot volume of the investigated spot in the sample channel normalized by the respective spot in the internal standard channel. The Heat/Filter-PDF spot ratio is then computed from these normalized values, yielding a highly reproducible result. Spot labels referring to Table 1 are shown underneath the respective bar chart. Error bars represent the standard deviation.

Supplemental Figure 3

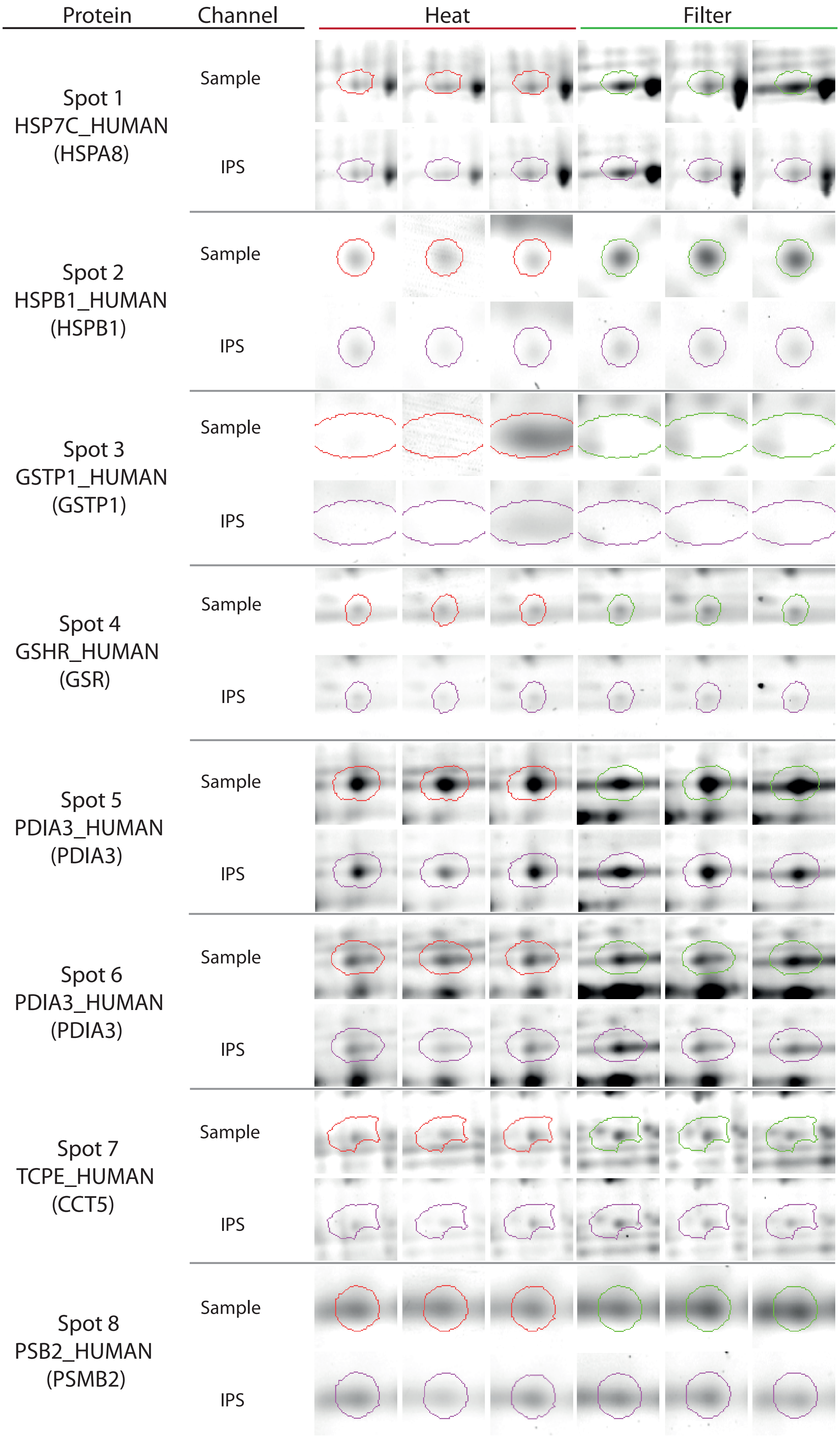

figure continued on next page

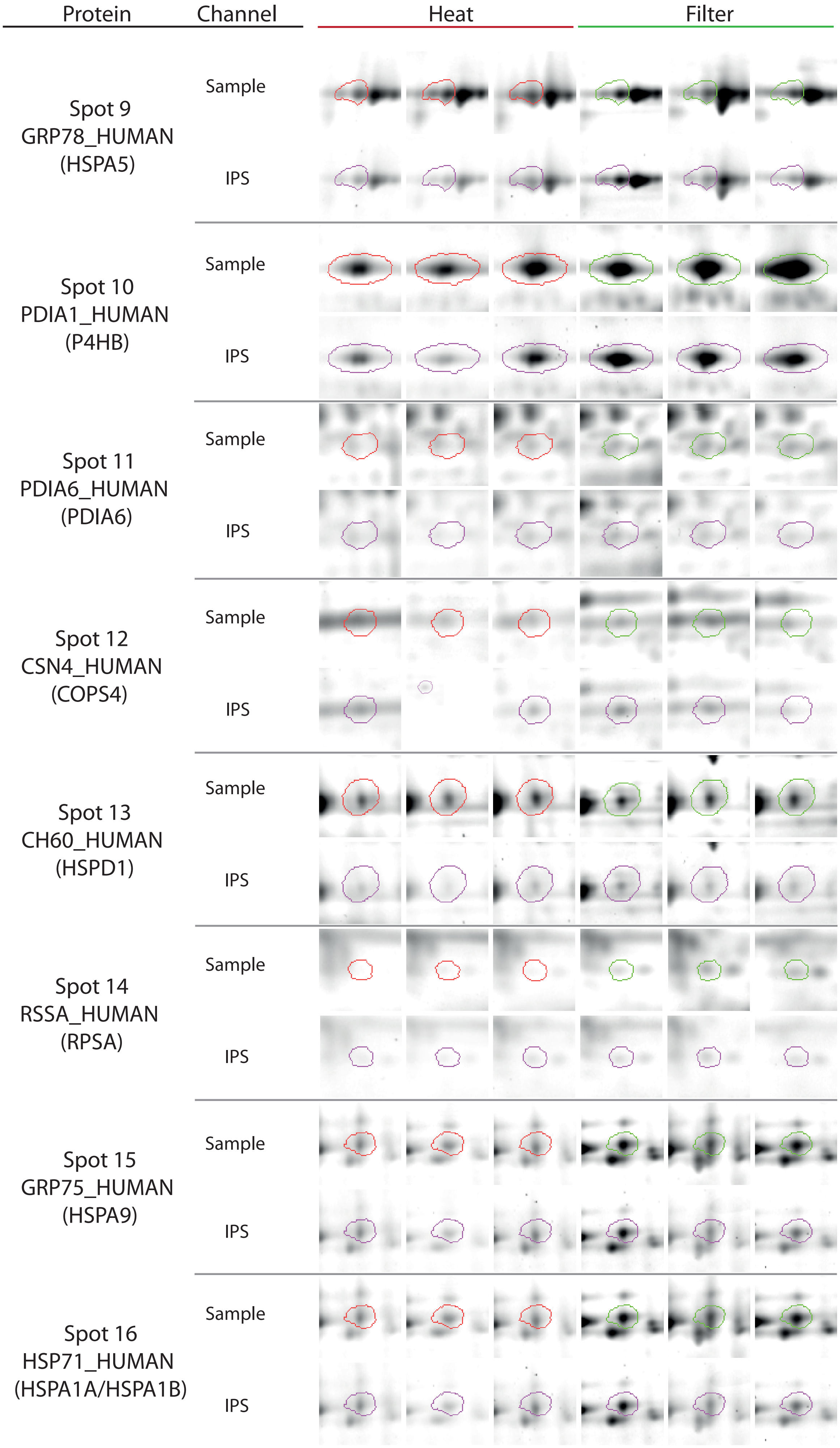

figure continued on next page

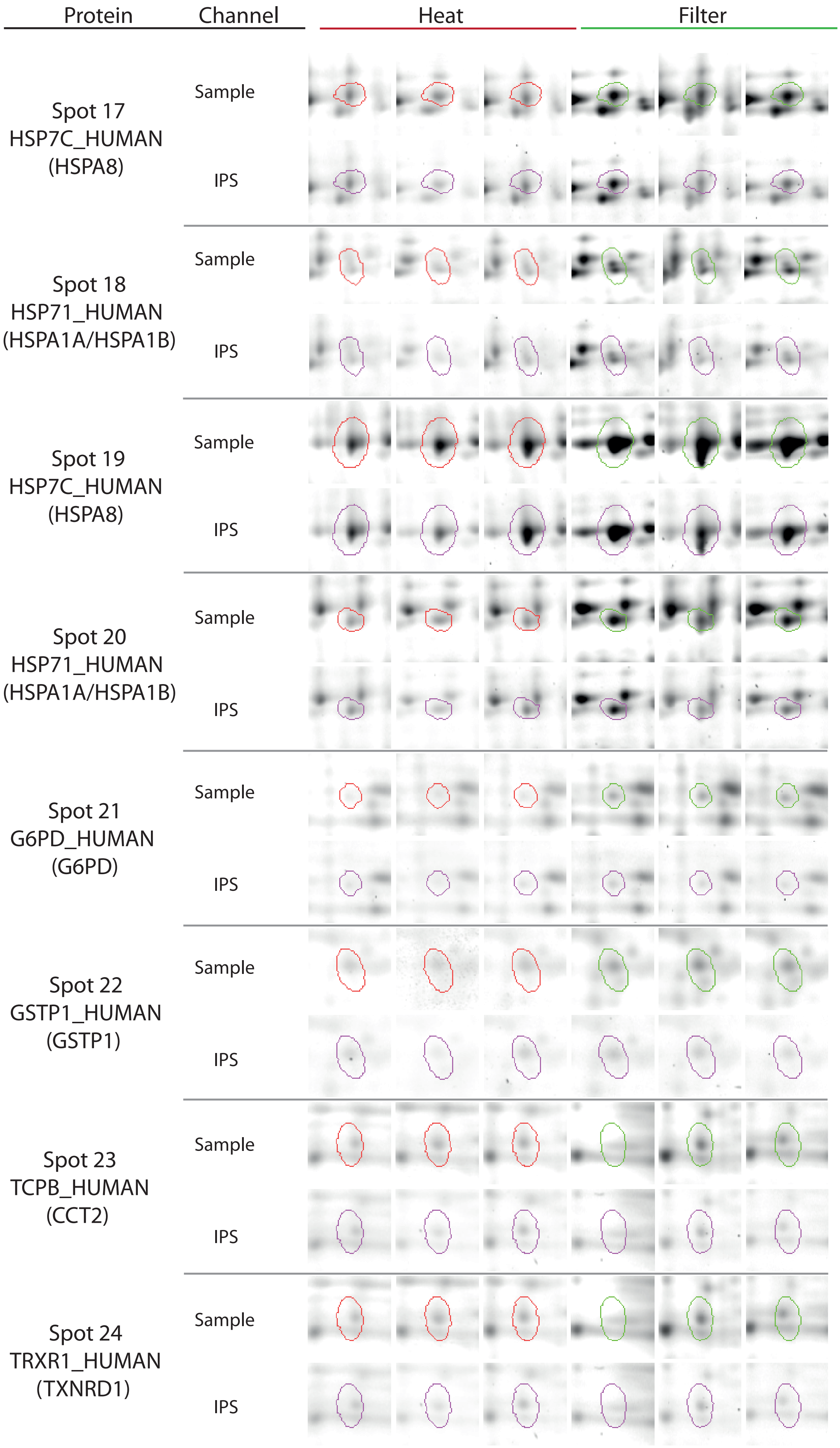

figure continued on next page

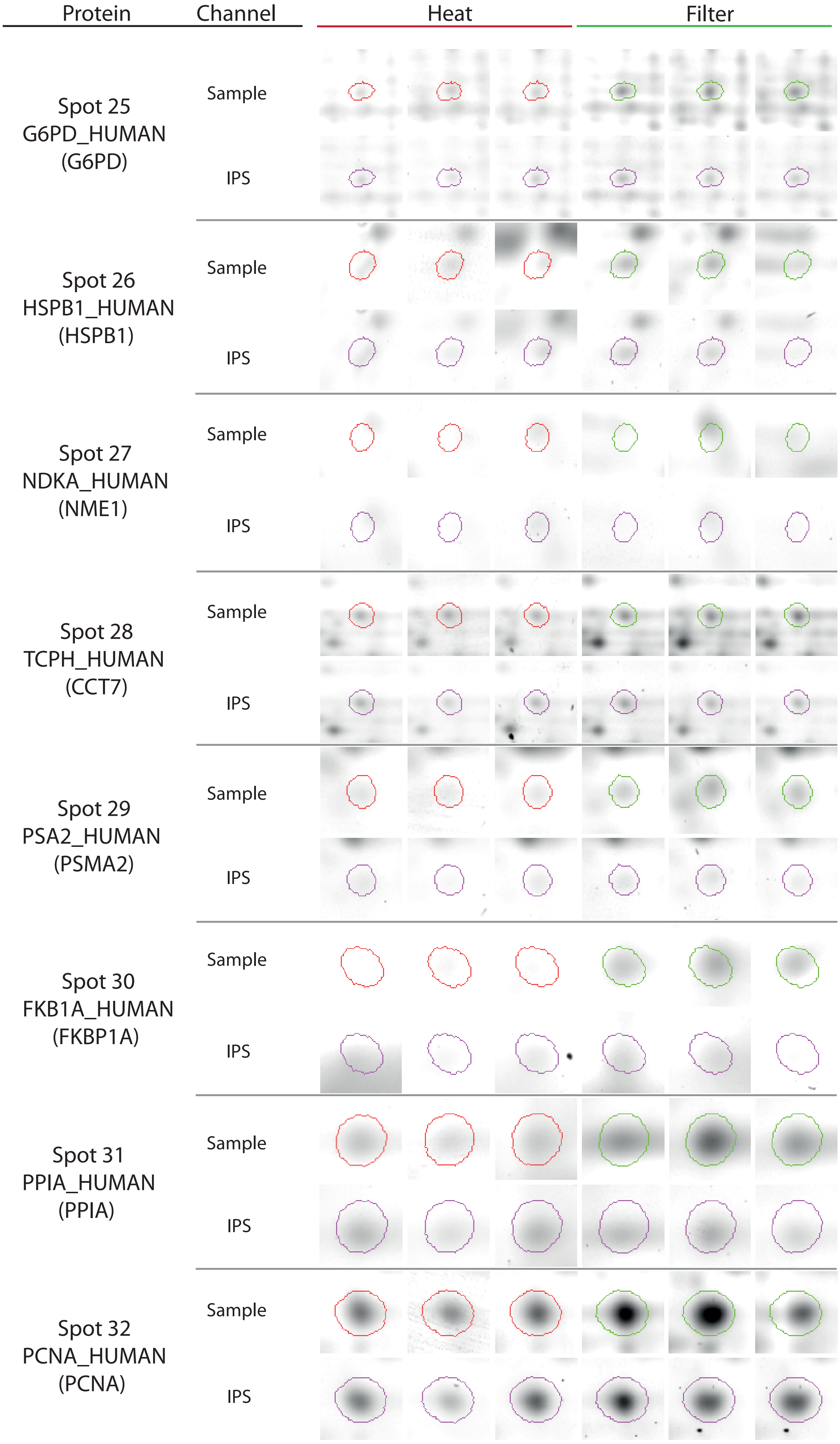

figure continued on next page

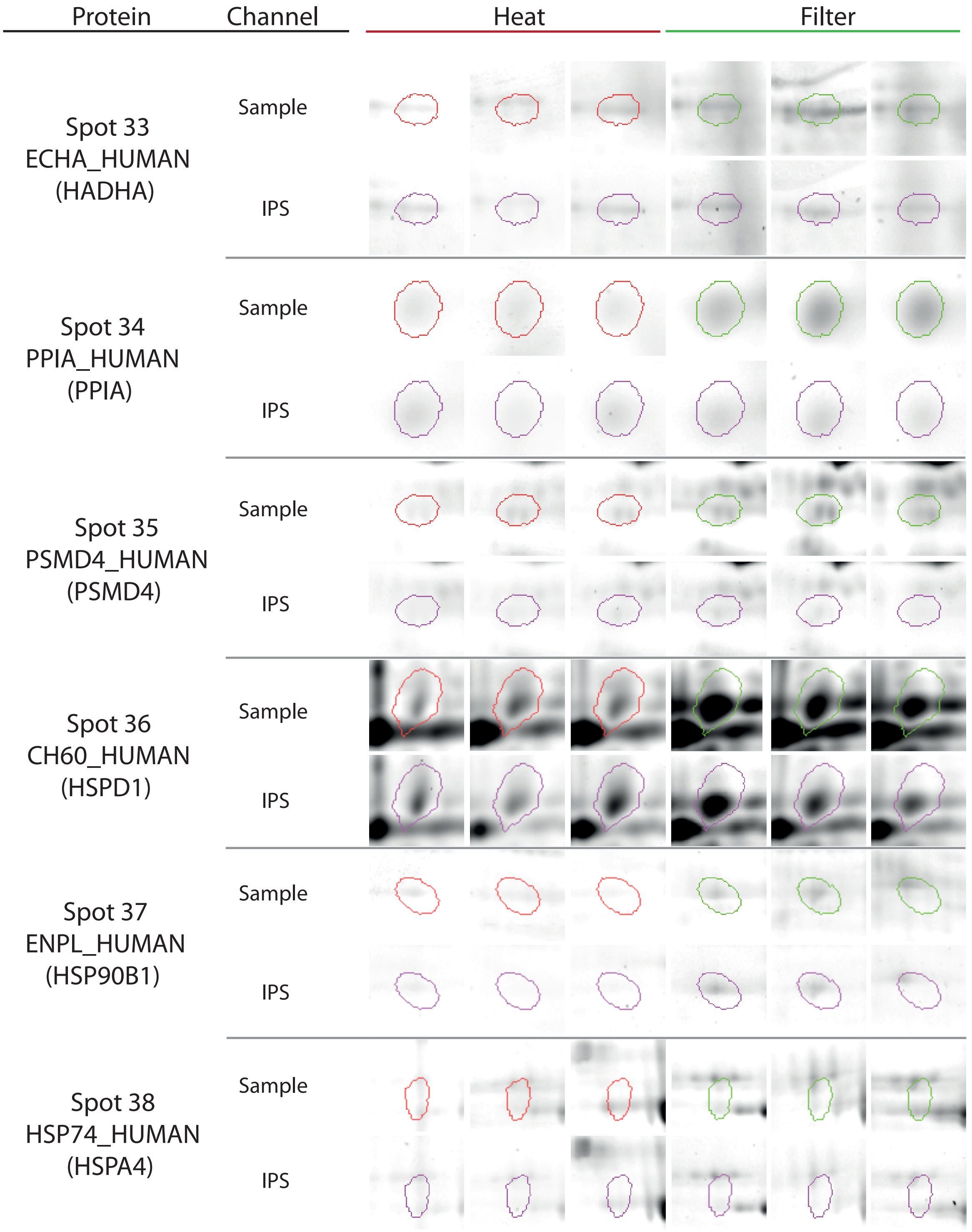
Protein
Channel
Heat
Filter
Spot 33
ECHA_HUMAN
(HADHA)
Sample
IPS
Spot 34
PPIA_HUMAN
(PPIA)
Sample
IPS
Spot 35
PSMD4_HUMAN
(PSMD4)
Sample
IPS
Spot 36
CH60_HUMAN
(HSPD1)
Sample
IPS
Spot 37
ENPL_HUMAN
(HSP90B1)
Sample
IPS
Spot 38
HSP74_HUMAN
(HSPA4)
Sample
IPS

Legend to Supplemental Figure 3: Details on investigated protein spots.

The column giving the spot label referring to Table 1 and the protein ID is followed individual spot images for each investigated spot in the two groups “Heat” (total protein lysates from mesothelial cells following exposure to H-PDF) and “Filter” (total protein lysates from mesothelial cells following exposure to F-PDF). For each spot the upper row contains the images of the Cy3 labeled sample channel, the lower row contains the respective region on the Cy5 labeled internal standard channel. The actual abundance of each DIGE replicate results from the relative spot volume of the investigated spot in the sample channel normalized by the respective spot in the internal standard channel.

Supplemental Table 1. Details on protein identifications in spots used for cross-omics comparison.

Label	*Protein Name*	*No. of peptides*[(a)]	*Mascot Score*[(b)]	*Sequence coverage (%)*	*Mr*[(c)]	*pI*[(d)]	*SwissProt entry name*	*SwissProt accession*	*Protein symbol*	*NCBI GeneID*	*EC No.*
1	Heat shock cognate 71 kDa protein	16	331	30	71055.3	5.37	HSP7C_HUMAN	P11142	HSPA8	3312	n/a
2	Heat shock protein beta-1	12	279	51	22825.5	5.98	HSPB1_HUMAN	P04792	HSPB1	3315	n/a
3	Glutathione S-transferase P	6	126	42	23341	5.43	GSTP1_HUMAN	P09211	GSTP1	2950	2.5.1.18
4	Glutathione reductase, mitochondrial	6	105	21	56220.9	8.78	GSHR_HUMAN	P00390	GSR	2936	1.8.1.7
5	Protein disulfide-isomerase A3	23	448	49	57145.9	5.98	PDIA3_HUMAN	P30101	PDIA3	2923	5.3.4.1
6	Protein disulfide-isomerase A3	24	420	57	57145.9	5.98	PDIA3_HUMAN	P30101	PDIA3	2923	5.3.4.1
7	T-complex protein 1 subunit epsilon	14	218	33	59632.8	5.44	TCPE_HUMAN	P48643	CCT5	22948	n/a
8	Proteasome subunit beta type 2	8	168	61	22821.6	6.52	PSB2_HUMAN	P49721	PSMB2	5690	3.4.25.1
9	78 kDa glucose-regulated protein precursor	5	102	11	72505.5	5.07	GRP78_HUMAN	P11021	HSPA5	3309	n/a
10	Protein disulfide-isomerase	23	525	46	57479.8	4.76	PDIA1_HUMAN	P07237	P4HB	5034	5.3.4.1
11	Protein disulfide-isomerase A6	13	247	46	48091.2	4.95	PDIA6_HUMAN	Q15084	PDIA6	10130	5.3.4.1
12	COP9 signalosome complex subunit 4	10	177	35	46239.6	5.57	CSN4_HUMAN	Q9BT78	COPS4	51138	n/a
13	60 kDa heat shock protein, mitochondrial	22	547	48	61187.4	5.7	CH60_HUMAN	P10809	HSPD1	3329	n/a
14	40S ribosomal protein SA	9	177	36	32833.4	4.79	RSSA_HUMAN	P08865	RPSA	3921	n/a
15	Stress-70 protein, mitochondrial	15	241	29	73929.9	5.81	GRP75_HUMAN	P38646	HSPA9	3313	n/a
16	Heat shock 70 kDa protein 1	19	388	38	70294.1	5.48	HSP71_HUMAN	P08107	HSPA1A/ HSPA1B	3303/ 3304	n/a
17	Heat shock cognate 71 kDa protein	16	331	30	71055.3	5.37	HSP7C_HUMAN	P11142	HSPA8	3312	n/a

18	Heat shock 70 kDa protein 1	24	475	49	70294.1	5.48	HSP71_HUMAN	P08107	HSPA1A/ HSPA1B	3303/ 3304	n/a
19	Heat shock cognate 71 kDa protein	17	268	37	71055.3	5.37	HSP7C_HUMAN	P11142	HSPA8	3312	n/a
20	Heat shock 70 kDa protein 1	25	423	54	70294.1	5.48	HSP71_HUMAN	P08107	HSPA1A/ HSPA1B	3303/ 3304	n/a
21	Glucose-6-phosphate 1-dehydrogenase	10	168	24	59218.9	6.39	G6PD_HUMAN	P11413	G6PD	2539	1.1.1.49
22	Glutathione S-transferase P	7	151	52	23341	5.43	GSTP1_HUMAN	P09211	GSTP1	2950	2.5.1.18
23	T-complex protein 1 subunit epsilon	13	215	31	57452.1	6.01	TCPB_HUMAN	P78371	CCT2	10576	n/a
24	Thioredoxin reductase 1, cytoplasmic	14	199	32	55470.2	6.07	TRXR1_HUMAN	Q16881	TXNRD1	7296	1.8.1.9
25	Glucose-6-phosphate 1-dehydrogenase	12	170	23	59675.2	6.39	G6PD_HUMAN	P11413	G6PD	2539	1.1.1.49
26	Heat shock protein beta-1	9	196	61	22825.5	5.98	HSPB1_HUMAN	P04792	HSPB1	3315	n/a
27	Nucleoside diphosphate kinase A	5	109	50	17137.6	5.81	NDKA_HUMAN	P15531	NME1	4830	2.7.4.6
28	T-complex protein 1 subunit eta	15	290	39	59798.1	7.55	TCPH_HUMAN	Q99832	CCT7	10574	n/a
29	Proteasome subunit alpha type-2	8	257	52	25996.3	6.92	PSA2_HUMAN	P25787	PSMA2	5683	3.4.25.1
30	FK506-binding protein 1A (Peptidyl-prolyl	5	115	53	12000.1	7.88	FKB1A_HUMAN	P62942	FKBP1A	2280	5.2.1.8
31	Peptidyl-prolyl cis-trans isomerase A	5	227	34	18229	7.68	PPIA_HUMAN	P62937	PPIA	5478	5.2.1.8
32	Proliferating cell nuclear antigen	4	117	27	29092.4	4.57	PCNA_HUMAN	P12004	PCNA	5111	n/a
33	Trifunctional enzyme subunit alpha, mitochondrial	21	277	31	83688.1	9.16	ECHA_HUMAN	P40939	HADHA	3030	4.2.1.17 / 1.1.1.211
34	Peptidyl-prolyl cis-trans isomerase A	5	227	34	18229	7.68	PPIA_HUMAN	P62937	PPIA	5478	5.2.1.8
35	26S proteasome non-ATPase regulatory subunit 4	12	144	40	40939.3	4.68	PSMD4_HUMAN	P55036	PSMD4	5710	n/a
36	60 kDa heat shock protein, mitochondrial	10	166	28	61187.4	5.7	CH60_HUMAN	P10809	HSPD1	3329	n/a

37	Endoplasmin, Heat shock protein 90 kDa beta member 1	26	523	33	92840.5	4.76	ENPL_HUMAN	P14625	HSP90B1	7184	n/a
38	Heat shock 70 kDa protein 4	28	600	47	95095.6	5.18	HSP74_HUMAN	P34932	HSPA4	3308	n/a

(a) Number of peptides matching in the peptide mass fingerprint based identification. (b) Mascot Protein Score: obtained from the combined (MS+MS/MS data) database search. (c) Relative molecular mass of the protein as calculated from the amino acid sequence of the polypeptide without any co- or posttranslational modifications. (d) Calculated *pI* of the polypeptide as obtained from SwissProt database.

Supplemental Table 2. Additional information on the analysis of the topic-defined microarray.

Biological process	***Overall frequency[a]***	***Overall percentage [b]***	***Observed number[c]***	***Expected number[d]***	***Enrichment factor[e]***
Immune response	40	3.2%	6	1.58	3.81
Angiogenesis	20	1.6%	3	0.79	3.81
Macrophage activation	51	4.1%	7	2.01	3.48
Protein complex assembly	22	1.8%	3	0.87	3.46
Protein complex biogenesis	22	1.8%	3	0.87	3.46
Neurological system process	36	2.9%	4	1.42	2.82
Cell-cell signaling	83	6.7%	9	3.27	2.75
Cellular defense response	56	4.5%	6	2.21	2.72
Response to stimulus	51	4.1%	5	2.01	2.49
Cell-cell adhesion	54	4.3%	5	2.13	2.35
Skeletal system development	33	2.7%	3	1.30	2.31
Immune system process	139	11.2%	12	5.48	2.19
Induction of apoptosis	110	8.8%	8	4.33	1.85
Negative regulation of apoptotic process	70	5.6%	5	2.76	1.81
Response to stress	112	9.0%	7	4.41	1.59
Apoptotic process	162	13.0%	10	6.38	1.57

All transcripts assessed on the topic defined microarray (total 1244) were queried using the PANTHER database for biological processes using the GO classification system. Observed numbers per biological process were normalized by the total number of transcripts. In comparison, transcripts which showed a Heat/Filter ratio of more than 2-fold, with a difference of more than two SD from 1.0, or a ratio of less than 0.5-fold, with a difference of more than two SD from 1.0 (total number of differentially abundant transcripts

was 236) were also queried for biological processes and the observed numbers were normalized by the number of transcripts used for the search. Only processes holding at least 3 transcripts observed as differentially abundant were included in the analysis. (a) Overall frequency of transcripts with the respective GO biological process assigned. (b) Proportion of transcripts assigned to a biological process as percentage. (c) Observed number of differentially abundant transcripts in the biological process. (d) Expected number of differentially abundant transcripts based on the proportion in all transcripts. (e) Enrichment factor for the process, comparing the expected and observed number of transcripts.
